# Comprehensive aberrations of proteasome complex in Alzheimer’s disease

**DOI:** 10.1002/alz.089220

**Published:** 2025-01-03

**Authors:** Shan Jiang

**Affiliations:** ^1^ Columbia University Medical Center, New York, NY USA

## Abstract

**Background:**

The ubiquitin‐proteasome system (UPS) is the primary protein degrading mechanism in eukaryotes, and is essential for cellular homeostasis. Dysregulation of the UPS has been linked to neurodegeneration through two hallmarks, pathogenic protein aggregation and aberrant proteostasis. However, the molecular changes that alter proteasome functioning in AD are poorly understood. It is also not known whether proteasome dysfunction is a cause or consequence of neurodegeneration. We hypothesized that proteasomes in the AD brain are affected across various levels, including functional attributes, proteomic profile, and transcriptional regulation.

**Method:**

To compare the activities of constitutive proteasome in AD patients samples with confounding factors (such as age and gender) matched controls, we used kinetics assays and native in‐gel activity assays. To compare the abundance changes of constitutive proteasome subunits in AD patients than in controls, we performed proteomics on gel‐isolated and purified proteasomes. To compare the RNA expression changes of constitutive proteasome subunits in AD patients than in controls, we interrogated RNA‐sequencing datasets from brain tissues and iPSC‐derived AD models.

**Result:**

We observed that AD patient samples displayed a decrease in the rate of substrate degradation in kinetics assays compared to the controls. This reduction in activity was further confirmed through native in‐gel activity assays. Proteomics analysis revealed decreased amounts of the proteasome subunits comprising the 20S and 19S particles, accompanied by a considerable proteasomal burden of phosphorylated tau in AD samples. Interrogation of RNA‐seq and snRNA‐seq datasets showed replicable down‐regulation of constitutive proteasome and chaperone genes in brain tissue from early Braak stages. Further analysis attributed this decrease mainly to neurons. Interrogation of RNA‐seq datasets of iPSC‐derived neurons and cerebral organoids showed down‐regulation of constitutive proteasome genes in both Abeta and Tauopathy models than in isogenic controls. Concomitantly, glial cells showed increases in the expression of these genes across Braak stages. The down regulation of assembly chaperones were reflective of the decreased rate of substrate degradation in kinetics assays in AD patients.

**Conclusion:**

Taken together, our results present not only the impairment of the constitutive proteasome in AD, especially in neurons, at RNA and protein expression level, but also the impairment of its functionality.

